# 4D MR imaging of cerebrospinal fluid flow in Chiari I malformation with and without syringomyelia and flow changes after decompressive surgery

**DOI:** 10.1186/1532-429X-14-S1-W1

**Published:** 2012-02-01

**Authors:** Jan Robert Kröger, Alena Juettner, Angela Brentrup, Barbara Fiedler, Gerard Crelier, Wolfram Schwindt, Thomas Niederstadt, Walter Heindel, David Maintz, Alexander C Bunck

**Affiliations:** 1 Department of Clinical Radiology, University Hospital of Muenster, Muenster, Germany; 2 Department of Neurosurgery, University Hospital of Muenster, Muenster, Germany; 3 Department of General Pediatrics, Subdivision Pediatric Neurology, University Hospital of Muenster, Muenster, Germany; 4 Institute for Biomedical Engineering, ETH and University of Zurich, Zurich, Switzerland

## Background

The aim of our study was to evaluate the feasibility of 4D phase contrast (PC) flow imaging for visualisation and analysis of cerebrospinal fluid (CSF) flow in Chiari I malformation. CSF flow was compared between healthy volunteers and patients with Chiari I malformation with and without syringomyelia. Moreover, the effect of a craniocervical decompression on CSF flow was studied in Chiari I patients with syringomyelia.

## Methods

20 patients with Chiari I malformation and 10 healthy volunteers were examined using 4D PC flow imaging. 11 patients had syringomyelia and 1 patient presented with a presyrinx. Of these, 9 patients underwent craniocervical decompression and flow data was assessed pre- and postoperatively. Flow images were analyzed by 2 blinded readers. Quantitative flow parameters including peak flow velocities, maximum flow volume and timing of systole were assessed at the craniocervical junction and the cervical spinal canal.

## Results

Qualitative analysis showed superior interreader agreement for 4D PC flow images compared to single-slice 2D axial (C1) and sagittal (mid-plane) images (κ = 0.67 vs. κ = 0.57 vs. κ = 0.02). Flow alterations were found in 17 patients consisting of anterolateral flow jet (n=14) or flow vortex (n=5) formation and flow inhomogeneity (n=3). In patients peak velocities at the craniocervical junction were found to be increased (-15.5 ± 11.3 cm/sec vs. -4.7 ± 0.7 in healthy volunteers, p < 0.001) with maximal velocities of up to 37.9 cm/sec. Of the 9 patients who underwent craniocervical decompression 4 showed a decrease in syrinx size postoperatively. Postoperatively, maximum flow volumes increased significantly at C2 and the lower levels of the cervical spine. In the group with regredient syrinx size peak flow velocities decreased (-19.4 ± 14.4 vs. -10.5 ± 3.8 p=0.07) while patients without change in syrinx size showed increased peak flow velocities postoperatively (-19.4 ± 14.4 vs. -25.7 ± 17.3 p=0.08).

## Conclusions

Analysis of cerebrospinal fluid dynamics using 4D PC flow imaging is superior to a 2D single slice approach. It allows for a comprehensive characterization of complex flow phenomena, which might be missed by 2D imaging. CSF flow is considerably altered in patients with Chiari I malformation. Analysis of CSF flow pre- and postoperatively suggests a correlation between reduction in peak flow velocity and change in syrinx size.

